# Signatures of Dimensionality and Symmetry in Exciton Band Structure: Consequences for Exciton Dynamics and Transport

**DOI:** 10.1021/acs.nanolett.1c02352

**Published:** 2021-08-31

**Authors:** Diana Y. Qiu, Galit Cohen, Dana Novichkova, Sivan Refaely-Abramson

**Affiliations:** † Department of Mechanical Engineering and Materials Science, Yale University, New Haven, Connecticut 06516, United States; ‡ Department of Molecular Chemistry and Materials Science, Weizmann Institute of Science, Rehovot 7610001, Israel

**Keywords:** Exciton band structure, Exciton dynamics, GW-BSE

## Abstract

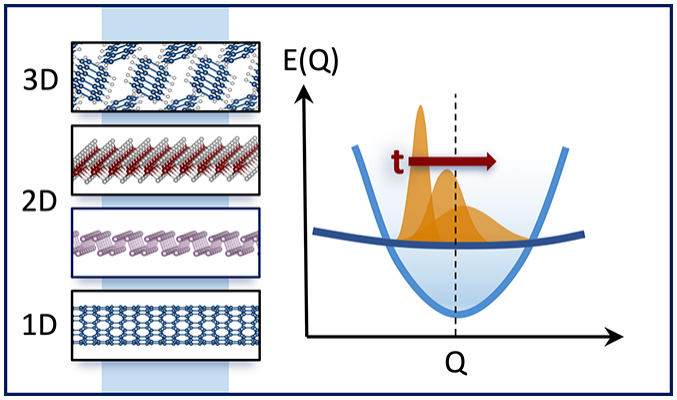

Exciton dynamics, lifetimes, and scattering are directly related to the exciton dispersion or band structure. Here, we present a general theory for exciton band structure within both *ab initio* and model Hamiltonian approaches. We show that contrary to common assumption, the exciton band structure contains nonanalytical discontinuities—a feature which is impossible to obtain from the electronic band structure alone. These discontinuities are purely quantum phenomena, arising from the exchange scattering of electron–hole pairs. We show that the degree of these discontinuities depends on materials’ symmetry and dimensionality, with jump discontinuities occurring in 3D and different orders of removable discontinuities in 2D and 1D, whose details depend on the exciton degeneracy and material thickness. We connect these features to the early stages of exciton dynamics, radiative lifetimes, and diffusion constants, in good correspondence with recent experimental observations, revealing that the discontinuities in the band structure lead to ultrafast ballistic transport and suggesting that measured exciton diffusion and dynamics are influenced by the underlying exciton dispersion.

In low-dimensional and nanostructured materials, the optical response is dominated by correlated electron–hole pairs—or excitons—bound together by the Coulomb interaction. By now, it is well-established that these large excitonic effects are a combined consequence of quantum confinement and reduced screening in low dimensions.^1−6^ However, many challenges remain in understanding the time evolution of these excitons, especially when it comes to correlating complex experimental signatures with underlying physical phenomena through the use of quantitatively predictive theories.

Recent advances in temporally and spatially resolved microscopies, such as transient absorption microscopy and time-resolved photoluminesence, allow direct observation of exciton relaxation and diffusion processes.^7−9^ For example, exciton diffusion in mono- and few-layer transition metal dichalcogenides (TMDs) reveals a ringlike diffusion pattern,^10^ while acene molecular crystals exhibit an asymmetric exciton diffusion.^11,12^ These diffusion processes reveal a wealth of competing scattering mechanisms and decay pathways, involving exciton–phonon interactions,^7,13,14^ exciton–exciton annihilation,^15−17^ occupation of dark states,^11,18,19^ electron–hole plasma,^20,21^ and environmental disorder.^20,22,23^

From a theoretical point of view, rate-equation approaches are typically used to model exciton diffusion, but these demand extensive parametrization,^11,24^ which hinders a predictive understanding of exciton structure–property relations. For near-equilibrium excitons in the low-field limit, *ab initio* Green’s function-based many-body perturbation theory, within the GW plus Bethe Salpeter equation (GW-BSE) approach,^25−33^ has been highly successful in predicting optical spectra across a wide variety of materials of different dimensionalities.^34,35^ GW-BSE methods can also give insight into exciton dynamics, including radiative recombination processes,^36−40^ multiexciton generation,^41−43^ and exciton–phonon interactions.^44−46^

Exciton dynamics require knowledge of the exciton band structure to accurately describe the phase space of momentum-conserving scattering processes. Until recently, the vast majority of theoretical approaches assumed that the exciton bands are either nondispersive^47^ or free-particle like.^48,49^ However, these assumptions neglect the energy splitting of longitudinal and transverse exciton modes.^50−52^ Furthermore, recent *ab initio* GW-BSE calculations of exciton band structure reveal that nonanalytic dispersion can arise in 2D materials,^53,54^ and bands of both positive and negative mass appear in the band structure of acene molecular crystals.^42,55^ Nonetheless, a general comprehensive understanding of how crystal symmetry and dimensionality affects exciton band structure and how signatures of that band structure manifest in exciton dynamics is still needed.

In this work, we explore signatures of symmetry and dimensional confinement in exciton band structures and the exciton time evolution. We use the GW-BSE method to compute exciton band structures of four prototypical systems of different dimensionality and symmetry. Remarkably, we find that the exciton band structure contains nonanalytical discontinuities at Γ in materials of all dimensions—a feature that is impossible to obtain from the electronic band structure alone. These discontinuities are a quantum phenomenon, arising from the exchange scattering of electron–hole pairs, and the degree of these discontinuities depends on the materials’ symmetry and dimensionality. Finally, we show that discontinuities in the exciton band structure can manifest in unexpected structure in the time evolution of an exciton wave packet, resulting in qualitative similarities to recent experiments and good agreement with experimental diffusion coefficients.

We begin by calculating the exciton band structure for four exemplary materials of different dimensionality and current interest: (1) the pentacene molecular crystal, a three-dimensional (3D) system with large excitonic effects;^56,57^ (2) mono- and bilayer MoS_2_, a quasi-two-dimensional (quasi-2D) system with degenerate excitons in different momentum-space valleys;^3,4^ (3) few-layer black phosphorus, a quasi-2D system with strongly bound excitons exhibiting linear dichroism;^58−61^ and (4) the (8,0) single-walled carbon nanotube, a prototypical quasi-one-dimensional (quasi-1D) system known to host large excitonic effects.^1,36,62^Figure 1 shows the calculated GW-BSE exciton band structure near **Q** = 0 for these four materials (see the Supporting Information (SI) for computational details). In all cases, ”bright” (i.e., dipole allowed) exciton bands exhibit nonanalytic behavior at the Γ point in the exciton band structure, while ”dark” exciton bands for which the transition from the ground state is dipole forbidden are parabolic. For simplicity, Figure 1 only shows the lowest-energy spin S = 0 (spin-singlet) dipole-allowed excitons and spin S = 0 dipole-forbidden excitons that are close in energy.

**Figure 1:**
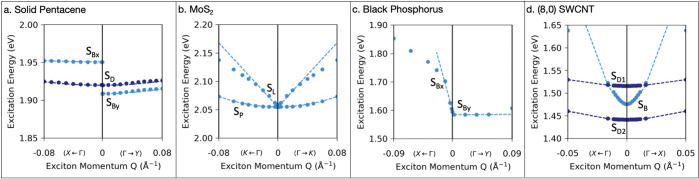
Exciton band structures for spin S = 0 excitons. Dots are GW-BSE results. Dashed lines show the fit to a model Hamiltonian. (a) Solid pentacene, with two exciton bands that are dipole-allowed (S_*B*_, light blue) and dipole-forbidden (S_*D*_, dark blue). (b) Monolayer MoS_2_ with excitons with linear (S_*L*_) and parabolic (S_*P*_) dispersion. (c) Monolayer black phosphorus with a dipole-allowed exciton with linear dispersion along the Γ to *X* direction. (d) (8,0) single-walled carbon nanotube, showing a nonparabolic low-lying exciton that is dipole-allowed (S_*B*_, light blue) and two nearby dark parabolic exciton bands (S_*D*1_ and S_*D*2_, dark blue).

To understand the source of the nonanalytic dispersion, we derive a model Hamiltonian fit to our *ab initio* results. In our *ab initio* calculations, electron–hole interactions are built on top of the quasiparticle (QP) picture by solving the BSE in the electron–hole basis,^32,33,63^ and the exciton band structure is obtained by solving the BSE at different exciton momenta **Q** following refs (53) and (64). The BSE is
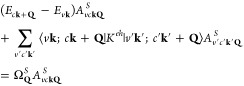
1Here, the index (*v***k**; *c***k** + **Q**) indicates a hole state |*v***k**⟩ and an electron state |*c***k** + **Q**⟩, where **k** is the crystal momentum and **Q** is the exciton center-of-mass momentum; *E*_*c***k**+**Q**_ and *E*_*v***k**_ are the QP energies calculated within the GW approximation; *S* indexes the exciton state at momentum **Q**; *A*_*v***k**;*c***k**+**Q**_^*S*^ is the amplitude of the free electron–hole pair; Ω_**Q**_^*S*^ is the exciton excitation energy; and *K*^*eh*^ is the electron–hole interaction kernel.

The interaction kernel in eq 1 is *K*^*eh*^ = *K*^*d*^ + *K*^*x*^. It consists of a direct term (*K*^*d*^), where the attractive interaction between the electron and the hole is mediated by the screened Coulomb interaction *W*, ⟨*v***k**; *c***k** + **Q**|*K*^*d*^|*v*^′^**k**^′^; *c*^′^**k**^′^ + **Q**⟩ = −∑_**GG′**_*M*_cc′_^*^(**k** + **Q**, **q**, **G**)*W*_**GG′**_(**q**)*M*_*vv′*_(**k**, **q**, **G**^′^), and an exchange term (*K*^*x*^), where the exchange scattering of an electron–hole pair gives rise to a repulsive term mediated by the bare Coulomb interaction v,
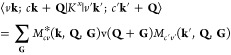
2Here, **G** are the reciprocal lattice vectors, **q** = **k** – **k**′, and *M* are the plane-wave matrix elements such that *M*_*nn*′_(**k**, **q**, **G**) = ⟨*n***k**|*e*^*i*(**q**+**G**)·**r**^|*n*′**k**′⟩.^31,33^ Note that the exchange term is unscreened as it arises from the variational derivative of the unscreened Hartree potential.^63^ We solve the BSE including both long- and short-range exchange.^33,64,65^

To derive a model Hamiltonian, we then expand the terms in the BSE (eq 1) within **Q**·**p** perturbation theory to find the behavior in the long wavelength limit. In this limit, the direct term goes as *K*^*d*^(**Q**) ∝–*Q*^2^ and results in an enhancement of the exciton effective mass.^53^ The exchange term goes as
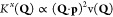
3where **p** = ⟨0|**r**|*S*(**Q**)⟩ is the dipole matrix element of the exciton state |*S*(**Q**)⟩ (see the SI). The Coulomb interaction in reciprocal space approaches different limits depending on the dimensionality (see the SI), leading to different dimension-dependent small **Q** (or long wavelength) limits of the exchange:
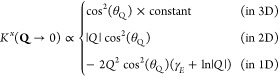
4where γ_*E*_ = 0.577 is the Euler constant^66^ and θ_**Q**_ is the angle between the exciton momentum, **Q**, and the matrix element, **p**. Thus, the behavior of the exchange term can vary dramatically with dimensionality and the anisotropy of the exciton’s dipole matrix element. The long wavelength component of the exchange term discussed above is only nonzero for excitons with a longitudinal component (i.e., **Q** ⊥̷ **p**).^50^ The longitudinal excitons correspond to those measured by the energy-loss function^64^ (see the SI) and may also be optically active in low-dimensional materials—when the polarization of the light has a component parallel to the momentum transfer in the crystal’s periodic direction^53^—and in bulk materials through interactions with the degenerate transverse polariton.^51,67^

Following eq 4, we can now derive a general form of the exciton dispersion in all dimensions (see the SI). In 3D, the exciton dispersion has the general form
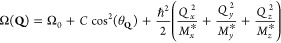
5where Ω_0_ is the excitation energy of the dipole forbidden exciton; Ω_0_ + *C* is the excitation energy of the dipole-allowed exciton; θ_**Q**_ is the angle of **Q** with respect to **p**; and M* is the exciton’s effective mass along *x*, *y*, *z* (see the SI).

With this understanding, we can interpret the dispersion of the pentacene crystal (Figure 1a). The exciton band structure shows two low-lying optically bright (*S*_*B*_) and dark (*S*_*D*_) spin-singlet states,^42,55^ with significant mass enhancement compared to the underlying quasiparticle band masses (see the SI). Additionally, the *S*_*B*_ band exhibits a discontinuous jump at **Q** = 0, which arises due to the asymmetry of the crystal. The absorption of light is only dipole allowed for polarizations along the **a**-axis (Γ → *X*). Thus, the longitudinal exciton, for which θ_**Q**_ = 0, has momenta along Γ → *X*, while the transverse exciton, for which θ_**Q**_ = 90°, has momenta along Γ → *Y*. The energy of the longitudinal branch is increased by a constant, *C* in eq 5, due to the long-range limit of the exchange *K*^*x*^, resulting in the discontinuity at **Q** = 0. This discontinuity is responsible for the longitudinal-transverse splitting in both exciton and polariton states^51^ and has been previously measured in bulk systems using EELS and two-photon spectroscopy.^67,68^ We note this splitting in the long wavelength limit, which was previously explored in phenomenological models,^50−52,69−71^ is a general phenomenon for optically bright excitons in 3D crystals exhibiting anisotropic light absorption. It can be understood independent of simplifying assumptions about the Wannier or Frenkel nature of the excitons. On the other hand, the dark singlet state *S*_*D*_ exhibits no such discontinuity because it is composed of dipole-forbidden electron–hole transitions, and consequently, the long-range exchange term is vanishingly small.

**Figure 2:**
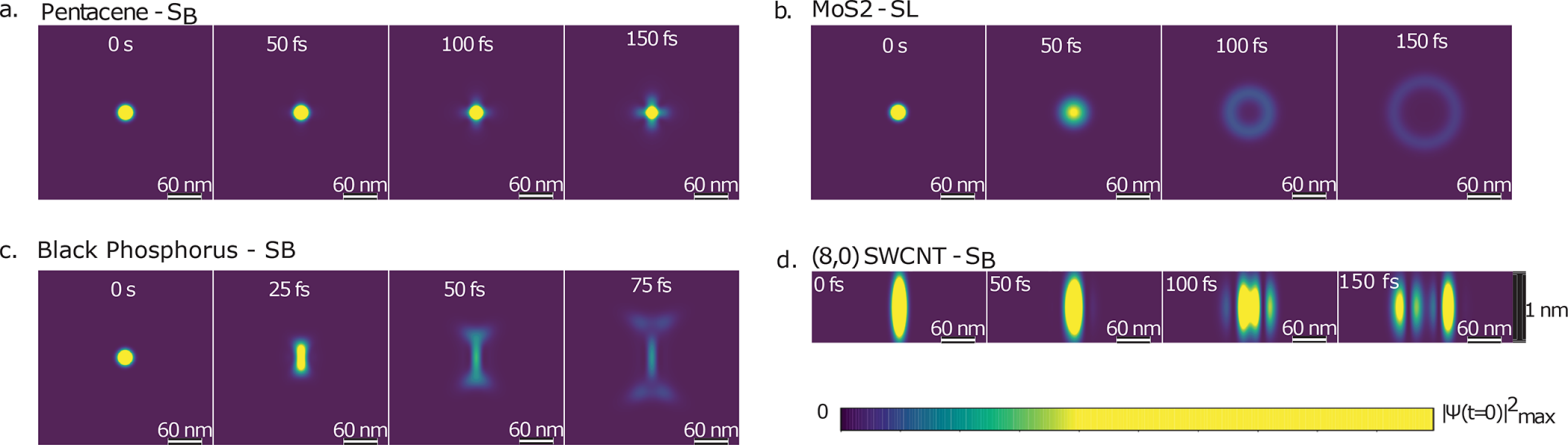
Time evolution of the amplitude squared (|Ψ(**R**, *t*)|^2^) of an initial Gaussian exciton wave packet as a function of the band structure of a single exciton band, as labeled in Figure 1, for (a) pentacene, (b) monolayer MoS_2_, (c) monolayer black phosphorus, and (d) the (8,0) SWCNT. The initial wave packet at *t* = 0 has the same spatial distribution in all cases, but the different band dispersion leads to distinct features in the wave packet evolution with time. In all cases, the distribution is normalized with respect to the maximum value at time *t* = 0.

Following eq 4, in 2D, the exciton dispersion of a nondegenerate exciton has the general form
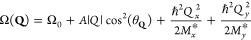
6where the |*Q*| term comes from the momentum-dependent behavior of the exchange interaction in 2D and can give rise to a linear dispersion that is nonanalytic at **Q** = 0 (see the SI). This general form is reflected in the calculated exciton dispersion of monolayer black phosphorus (Figure 1c). For the lowest energy singlet exciton, the dispersion is linear along the Γ to *X* direction, while the dispersion is parabolic along the Γ to *Y* direction due to the crystalline asymmetry, where optical absorption is only allowed for polarizations along the Γ to *X* direction. Thus, the long-range exchange interaction is zero for excitons with center-of-mass momentum, **Q**, along the Γ to *Y* direction perpendicular to the exciton dipole matrix element (i.e., the transverse excitons) but goes as |*Q*| for excitons with center-of-mass momentum, **Q**, parallel to the exciton dipole matrix element (Γ to *X*) (i.e., the longitudinal excitons). As the thickness of black phosphorus is increased from one layer to two, three, and four layers, the linearly dispersing region becomes restricted to a smaller region of **Q**-space, inversely proportional to the material thickness (see the SI). Thus, as the number of layers increases, we expect to recover the 3D limit, where we see a jump discontinuity between the longitudinal and transverse bands.

In monolayer MoS_2_ (Figure 1b), the picture changes due to the 2-fold degeneracy of the lowest energy exciton at **Q** = 0. As previously reported in ref (53), the interplay of intervalley and intravalley exchange in MoS_2_ gives rise to two optically bright low-energy exciton bands that are degenerate at **Q** = 0. In the upper band the intervalley and intravalley exchange add coherently, leading to a massless v-shaped dispersion, while in the lower band the intervalley and intravalley exchange cancel, leaving a parabolic dispersion whose effective mass is enhanced by the large exciton binding energy. In the small **Q** limit, the dispersion of the linear band (*S*_*L*_) to lowest order in **Q** follows Ω(**Q**) = Ω_0_ + 2*A*|*Q*|, where *A* is a constant. The parabolic band (*S*_*P*_) follows . We thus see that in quasi-2D the long-range limit of the exchange in the electron–hole basis (eq 2) allows for the possibility of linear dispersion, while the dipole selection rules along different directions, combined with the excitonic mixing of different valleys, determines how a given excitonic band will disperse independent of the dispersion of the underlying quasiparticle bands. Unlike in 3D, there is no splitting of the longitudinal and transverse modes in quasi-2D, and the purely longitudinal mode can be optically active, since the polarization of the external field is not constrained to be parallel to the exciton dipole matrix element (see the SI). Intriguingly, if we extend our analysis to bilayer MoS_2_, we now have an effective Hamiltonian with four degenerate excitons at **Q** = 0 along with the interplay of intervalley, intravalley, interlayer, and intralayer exchange. In three solutions, the exchange interactions cancel, while in the fourth solution, the exchange interactions add coherently, giving rise to three parabolic exciton bands and one nonanalytic v-shaped band all degenerate at **Q** = 0 (see the SI). In fact, any centrosymmetric system with *N*-fold degenerate excitons at **Q** = 0 should have *N* – 1 parabolic bands and one v-shaped band due to the symmetry of the exchange matrix, while noncentrosymmetric crystals with *N*-fold degenerate excitons will in general have *N* linearly dispersing bands for all cases except *N* = 2 (see SI).

In 1D, following eq 4, the exciton dispersion has the general form
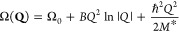
7where now the long-range exchange introduces a term that goes as *Q*^2^  ln |*Q*|.^72^ This behavior is reflected in the dispersion of excitons in the quasi-1D (8,0) SWCNT (Figure 1d). This system contains a large number of strongly bound spin-singlet excitons,^1,62^ with several optically dark states below and around the first bright peak at ∼1.5 eV.^36^ For simplicity, we focus on the exciton states close in energy to the lowest energy bright exciton. Here, the quasi-1D confinement leads to two types of very different low-lying singlet states. The dispersion of the optically bright state *S*_*B*_ fits well to the expression in eq 7. In contrast, the two nearby dark states, *S*_*D*1,2_, have a parabolic dispersion with relatively large effective masses (see the SI). In 1D, as in 2D, the exchange contributes to both longitudinal and transverse modes, and the purely longitudinal mode can be optically active, as is the case in the (8,0) SWCNT.

Next, we examine how signatures of symmetry and dimensionality in the exciton band structure manifest in the exciton dynamics. Here, we consider the semiclassical transport of an exciton wave packet in the ballistic limit. In reality, one does not excite a single exciton eigenmode but rather a wave packet of excitons, which may have a Gaussian profile in real-space. In reciprocal space, the time evolution of such a Gaussian wave packet follows
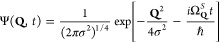
8, where Ω_**Q**_^*S*^ is the exciton dispersion and σ is the standard deviation of the distribution in reciprocal space. We chart the real-space evolution of the exciton wave packet by taking the Fourier transform of the time evolution of the initial Gaussian reciprocal-space wave packet. Figure 2 shows the resulting time evolution for the examined excitons in the studied systems. To isolate the role of the band structure independent of confounding factors, we first examine very short propagation times on the order of exciton decoherence times, ∼100 fs. Our results show an immediate connection between the dimension-specific exciton band structure features discussed above and the wave packet evolution.

Naively, we expect a Gaussian wave packet to remain Gaussian, but this is purely a consequence of the parabolic energy dispersion, which gives the time evolution the general form of a complex Gaussian. Thus, in all cases, the parabolic exciton bands maintain a Gaussian shape, and for the short time scales considered, they are well-approximated as completely nondispersive (see the SI). Conversely, the nonparabolic bright exciton bands result in unexpected patterns in the wave packet time evolution. In pentacene, the strong anisotropy leads to a crosslike shape in the evolution of the wave packet with an average velocity of 0.2 nm/fs. In MoS_2_, the linear band forms a ringlike shape, as the wave packet quickly propagates away from the cusp at **Q** = 0, resembling recent experimental observations.^10^ The average velocity of the wave packet is 0.4 nm/fs. A similar effect occurs in black phosphorus, but only along the direction of linear dispersion. The average velocity is 0.9 nm/fs. Finally, in the (8,0) SWCNT, the nonparabolic band leads to an oscillating pattern as the wave packet disperses along the nanotube, with an average velocity of 0.25 nm/fs. Thus, even at very short time scales before the onset of diffusive behavior, the initial femtoseconds of the exciton time evolution can set up an unexpected spatial distribution, due to the fast quasi-ballistic transport, which establishes the initial conditions of the exciton diffusion. However, we can extend our analysis to the diffusive regime by considering the relation between diffusivity and velocity within the relaxation time approximation. In TMDs, if we take the previously reported upper limit on the coherence lifetime of 10–30 fs^73^ as the relaxation time, the computed velocities lead to a diffusion coefficient of 8–24 cm^2^/s, in decent agreement with experimental values ranging between 0.3 and 15 cm^2^/s for free-standing and encapsulated WS_2_ and WSe_2_ monolayers,^10,16,18,20,74^ where the magnitude of the exchange interaction is similar to that in MoS_2_.^75^ For pentacene, we use our previous work^42^ to estimate an upper bound on the relaxation time of 30–70 fs, leading to an upper-bound on the diffusion coefficient of 6–14 cm^2^/s, again on the same order as experimental values ranging between 0.8 and 3 cm^2^/s in tetracene and pentacene crystals.^11,12,76^

Finally, we discuss how the signatures of the exciton band structure are expected to manifest in the exciton radiative lifetimes, following refs (36) and (72). We find that the linear dispersion in 2D has the effect of shortening the radiative lifetime at room temperature by as much as a factor of 10 by decreasing the density of states to which the exciton can thermalize. For MoS_2_, the calculated radiative lifetime, considering only intravalley excitons, at 300 K is 570 ps, in the range of experimentally reported values,^37,77,78^ and increases to 1.03 ns when thermalization to the intervalley dark state is included. The calculated radiative lifetime of the (8,0) SWCNT is 0.2 ps, compared to 1 ps when the *ab initio* exciton band structure is neglected.^36^ In general, the nonanalytic exciton dispersion in low-dimensions results in a steeper dispersion curve and shorter radiative lifetimes, while excitonic mass enhancement of the parabolically dispersing excitons will tend to enhance the radiative lifetime. Likewise, we expect that the nonanalytic exciton dispersion will enhance exciton–phonon scattering lifetimes, since the nonanalytic dispersion decreases the density of states at the exciton band edge. The *ab initio* exciton band structure is further expected to influence exciton–exciton scattering processes, e.g. singlet fission in molecular crystals,^42,79,80^ where the key metric is the availability of triplet states at half the singlet excitation energy.

In conclusion, we have performed state-of-the-art first-principles calculations to realize the relation between exciton band structure and materials’ symmetry and dimensionality. We show that the long-range exchange interaction gives rise to exciton dispersions that have nonanalytic discontinuities in the small-**Q** limit. In 3D, crystal asymmetry giving rise to linear dichroism leads to a jump discontinuity in the dipole-allowed exciton bands. In quasi-2D, the exciton has a massless nonanalytic dispersion when the exciton momentum has a component parallel to the exciton dipole matrix element. In quasi-1D, there is a removable discontinuity in the dispersion of the bright states at **Q** = 0. Qualitatively, the discontinuity in the exciton dispersion can be understood as an effect arising from the long-range dipole–dipole interactions due to the exciton’s own polarization field, mathematically analogous to longitudinal and transverse splitting of optical phonons.^52^ We show that the nonparabolic exciton dispersion has consequences for initial quasi-ballistic transport of the exciton, leading to unexpected patterns in the time evolution of an exciton wave packet and diffusion coefficients in good agreement with experiment, and should thus be considered in a complete picture of exciton dynamics.

## Supporting Information Available

(1) Computational details; (2) layer-dependence of exciton band structures for MoS_2_ and black phosphorus; (3) derivation of effective Hamiltonians for small **Q**; (4) parameters for fitting the model Hamiltonian; (5) discussion of longitudinal and transverse excitons in the BSE kernel; (6) EELS spectra for pentacene (PDF)

## Notes

D.Y.Q. and S.R.A. share equal correspondence of this work.
