# Supplementary material for: Signatures of Dimensionality and Symmetry in Exciton Band Structure: Consequences for Exciton Dynamics and Transport
Source: Nano Lett. 2021 Aug 31;21(18):7644–50. doi: 10.1021/acs.nanolett.1c02352 (PMC8890683; doi:10.1021/acs.nanolett.1c02352)
Supplement: Supplementary file 1 — nl1c02352_si_001.pdf [file nl1c02352_si_001.pdf]

Supplemental Information: Signatures of Dimensionality and Symmetry in Exciton Bandstructure: Consequences for Exciton Dynamics and Transport

Diana Y. Qiu,[1,*] Galit Cohen,[2] Dana Novichkova,[2] and Sivan Refaely-Abramson[2,†]

[1]*Department of Mechanical Engineering and Materials Science, Yale University New Haven, CT 06516, USA*

[2]*Department of Molecular Chemistry and Materials Science, Weizmann Institute of Science, Rehovot 7610001, Israel*

I. COMPUTATIONAL DETAILS

We first calculate the mean-field wavefunctions and electronic bandstructure within density-functional theory (DFT) using the Quantum Espresso code[7]. The GW quasiparticle (QP) energies and the Bethe Salpeter equation (BSE) excitations for each center-of-mass momentum $\mathbf{Q}$ of the excitons are then computed using the BerkeleyGW code[5]. The QP bandstructure, the transitions dominating the exciton dispersion around $\mathbf{Q} = 0$, and the exciton bandstructures for all examined systems are shown in Fig. S1.

A. Pentacene Crystal

For the pentacene crystal, we use the experimentally reported bulk structure PENCEN08[26] as given in the Cambridge Crystallographic Data Centre (CCDC)[1]. We use experimental lattice parameters, although similar results can be achieved by performing a DFT-based structural optimization[19,25]. The DFT starting point was computed using the Perdew-Burke-Ernzerhof (PBE) exchange-correlation functional[15]. The GW calculation was done on a $8 \times 8 \times 4$ k-point grid, with a plane-wave basis and norm-conserving pseudopotentials with a 60 Ry wave function cutoff. We used a 7 Ry cutoff for the dielectric matrix and included 499 Kohn-Sham states in the sum over empty states. Dynamical screening effects were accounted for with the Hybertsen-Louie Generalized Plasmon Pole model (HL-GPP)[9]. The BSE was solved for each center-of-mass exciton momentum $\mathbf{Q}$ using four occupied and four empty bands, for both singlet and triplet states, with the interaction kernel evaluated on a $8 \times 8 \times 4$ Monkhorst-Pack k-point grid.

B. Monolayer MoS_2

For MoS_2, we first relaxed the structure using DFT in the local density approximation (LDA)[12] using a 350 Ry planewave cutoff. This results in a lattice constant of $3.15\AA$, which deviates less than 1% from the experimental lattice constant of few-layer MoS_2[29]. The semicore $4s$ and $4p$ states were included as valence states in the pseudopotential, and the wavefunctions were calculated using a 125 Ry plane wave cutoff, which converges the bare exchange within 5 meV.

The QP bandstructure was calculated using the GW_0 method, with the eigenvalues of the Green's function (G) updated until the QP energies converged within 50 meV. Dynamical screening effects were accounted for with the Hybertsen-Louie Generalized Plasmon Pole model (HL-GPP)[9]. The supercell contained $25\AA$ of vacuum in the aperiodic direction, and a truncated Coulomb interaction[10] was used to prevent spurious interactions between periodic images. To converge the QP energies within 100 meV, the dielectric matrix energies were calculated on a $24 \times 24 \times 1$ k-grid with an energy cutoff of 35 Ry for the dielectric matrix and $6,000$ bands included in the summation over empty states.

For each $\mathbf{Q}$, the BSE was solved on a $168 \times 168 \times 1$ k grid, which converges the absolute QP excitation energies within 100 meV and the relative energies within 5 meV at $\mathbf{Q} = 0$ and $\mathbf{Q} = K$. We scanned over twenty values of $\mathbf{Q}$ and $-\mathbf{Q}$ between $0.001a^{-1}$ and $0.04a^{-1}$, where a^{-1} is the inverse lattice constant. Spin-orbit coupling was included as a perturbation in the QP bandstructure. For the BSE, QP bands were indexed by the expectation value of S_z at the K point in a fully relativistic calculation. Then, the BSE was solved separately for like- and unlike-spin transitions between bands with different values of S_z at K.

C. Monolayer Black Phosphorus

For black phosphorus, we first relaxed the geometry in a supercell arrangement using a plane wave basis with norm conserving PBE[16] pseudopotentials with a van der Waals correction[8], a 55 Ry wave function cutoff, and uniform $28 \times 20 \times 1$ k points sampling. The functional and form of the van der Waals correction were selected by comparing relaxed structural parameters of bulk black phosphorus with experimental values following our previous work in Ref.[18]. A large vacuum was included between repeated supercells in the aperiodic (c-axis) direction so that 99% of the charge density was contained in half of each supercell.

We performed a one-shot GW calculation to obtain the QP bandstructure. The dynamical screening effects were accounted for with the Hybertsen-Louie generalized plasmon pole (HL-GPP) model[9]. The dielectric matrix was calculated on a $7 \times 5 \times 1$ uniform q grid with an additional 10 q-points in the small-q region, following the Nonuniform Neck Subsampling (NNS) method[2], which is equivalent to a uniform sampling of $70 \times 50 \times 1$ q points. The dielectric matrix included plane-wave components up to 15 Ry, and unoccupied states with energy up to 10 Ry were included in the sum over unoccupied states in the calculation of both the polarization and the GW self energy. These parameters converge the QP gap at Γ to better than 0.1 eV. The static remainder technique was used to speed up convergence with respect to unoccupied states[4], and a truncated Coulomb interaction was used to prevent spurious interactions between periodic images[10].

For each **Q**, The BSE matrix elements were calculated on a uniform $28 \times 20 \times 1$ k grid and then interpolated to a $160 \times 160 \times 1$ fine k grid using the Clustered Sampling Interpolation (CSI) technique[2]. In CSI, BSE matrix element are explicitly calculated for 10 additional k points in each cluster, sampled along the (110) direction. This converged the BSE eigenvalues to better than 0.1 eV.

D. (8,0) Single-Walled Carbon Naotube

For (8,0) SWCNT, we used the structural parameters taken from Ref.[27], with a tube radius of 6.31 Å. The DFT starting point was computed using the local density approximation (LDA) functional[12]. The GW calculations were done on a $32 \times 1 \times 1$ k-point grid, with a plane-wave basis and norm-conserving pseudopotentials with a 60 Ry wave function cutoff. We used a 12 Ry cutoff for the dielectric matrix and included 571 Kohn-Sham states in the sum over empty states. Dynamical screening effects were accounted for with the Hybertsen-Louie Generalized Plasmon Pole model (HL-GPP)[9], and a truncated Coulomb interaction[10] was used to prevent spurious interactions between periodic images. The BSE was solved for each center-of-mass momentum **Q** of the excitons using 11 occupied and 10 empty states, with the interaction kernel evaluated on a $256 \times 1 \times 1$ Monkhorst-Pack k-point grid.

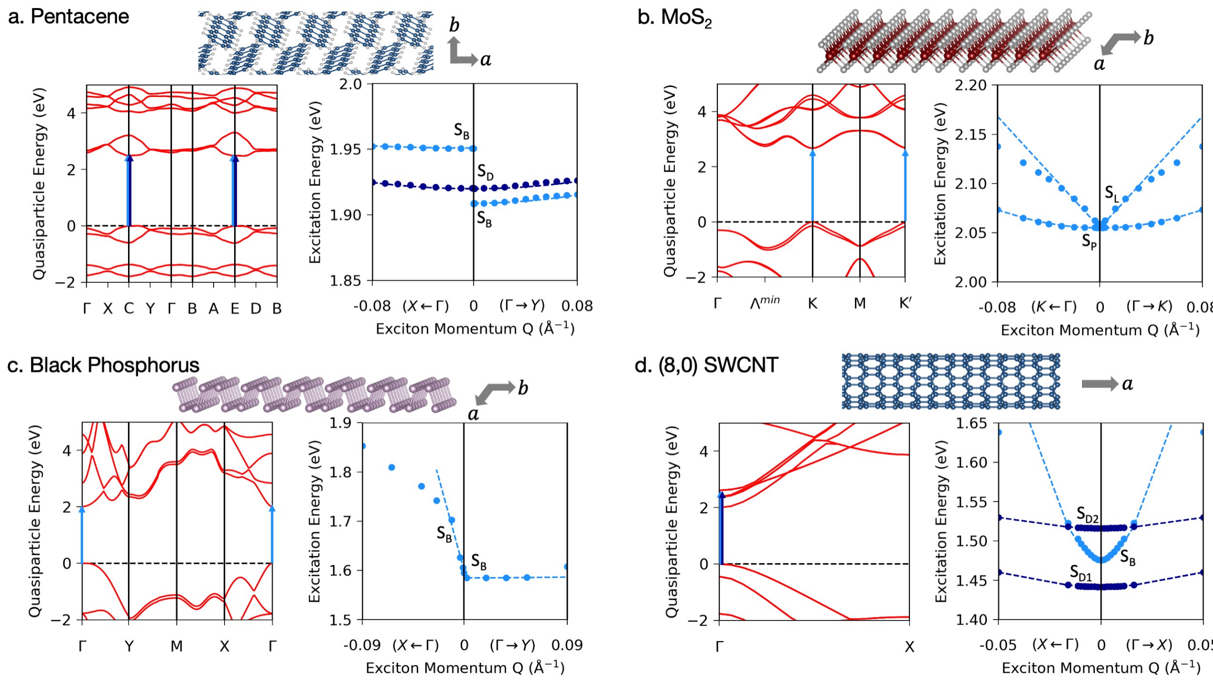

FIG. S1. GW-BSE results for the quasiparticle (left) and exciton (right) bandstructure, as well as atomic structures of the systems examined in this work: (a) pentacene molecular crystal; (b) monolayer MoS_2; (c) 2D Black Phosphorus; and (d) (8,0) single-walled carbon nanotube. Arrows represent the $\mathbf{Q} = 0$ excitons studied for each system, and the exciton dispersion functions used for dynamics are computed for small finite $\mathbf{Q}$ around these excitations.

II. LAYER-DEPENDENCE OF QUASI-2D EXCITON BANDSTRUCTURES

A. Bilayer MoS_2

At $\mathbf{Q} = 0$, bilayer MoS_2 has four nearly degenerate low-energy excitons corresponding to the degeneracy of the K and K' valleys and the degeneracy of the two layers. Fig. S2 shows the exciton bandstructure near Γ for the spin S=0 exciton states arising from transitions between like-spin bands and the spin S=1 exciton states arising from transitions between unlike-spin bands. The S=1 excitons consist of four nearly degenerate parabolic bands, and the S=0 excitons consist of two degenerate parabolic bands, one parabolic band with a smaller band mass, and one non-analytic v-shaped band.

To understand this dispersion, we derive a model Hamiltonian by expanding the exciton states within $\mathbf{Q} \cdot \mathbf{p}$ perturbation theory. The basis consists of four nearly degenerate exciton states: one

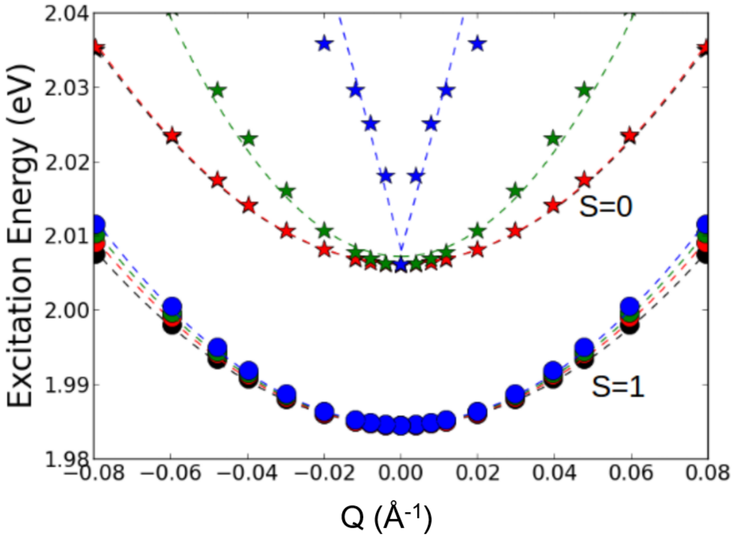

FIG. S2. Exciton bandstructure of freestanding bilayer MoS_2 calculated within GW-BSE. Circles are excitons arising from transitions between unlike-spin bands (S=1), and stars correspond to excitons arising from transitions between like-spin bands (S=0). The dashed lines are fit to a model Hamiltonian.

intralyer exciton at K ($|S_1\rangle$), one interlayer exciton at K ($|S_2\rangle$), one intralayer exciton at K' ($|S_3\rangle$), and one interlayer exciton at K' ($|S_4\rangle$). Since these states are nearly degenerate, we assume that the direct and exchange matrix elements on the diagonal are approximately the same for all four excitons. Thus, the model Hamiltonian has the approximate form

$$H^{BSE} = \mathbf{\Omega}_0 + \begin{bmatrix} A|Q| + \alpha Q^2 & A'|Q| & A'''|Q| + \alpha' Q^2 & A''|Q| \\ A'|Q| & A|Q| + \alpha Q^2 & A''|Q| & A'''|Q| + \alpha' Q^2 \\ A'''|Q| + \alpha' Q^2 & A''|Q| & A|Q| + \alpha Q^2 & A'|Q| \\ A''|Q| & A'''|Q| + \alpha' Q^2 & A'|Q| & A|Q| + \alpha Q^2 \end{bmatrix}. \quad (1)$$

Here, $\mathbf{\Omega}_0$ is a diagonal matrix containing the exciton excitation energies at $\mathbf{Q} = 0$; A is the intralayer intravalley exchange; A' is the interlayer intravalley exchange; A'' is the interlayer intervalley exchange; A''' is the intralayer intervalley exchange; α is the intralayer intravalley direct interaction; and α' is the intralayer intervalley direct term. We assume that the interlayer direct term is strictly zero, since the overlap of the conduction band wavefunction is small.

Solving this Hamiltonian gives four solutions

$$\begin{aligned}
\Omega_1(\mathbf{Q}) &= \Omega_0 + (\alpha - \alpha')Q^2 + (A + A' - A'' - A''')|Q| \\
\Omega_2(\mathbf{Q}) &= \Omega_0 + (\alpha - \alpha')Q^2 + (A - A' + A'' - A''')|Q| \\
\Omega_3(\mathbf{Q}) &= \Omega_0 + (\alpha + \alpha')Q^2 + (A - A' - A'' + A''')|Q| \\
\Omega_3(\mathbf{Q}) &= \Omega_0 + (\alpha + \alpha')Q^2 + (A + A' + A'' + A''')|Q|
\end{aligned} \tag{2}$$

We make the further assumption that the magnitude of all of the exchange terms are roughly the same: i.e., $A = A' = A'' = A'''$. This is reasonable since the exchange matrix elements term depend on the overlap of the electron and hole wavefunctions within the same exciton. This overlap must be similar for all four states, since these states are degenerate at $\mathbf{Q} = 0$. Then, the dispersion of the four states simplifies to:

$$\begin{aligned}
\Omega_1(\mathbf{Q}) &= \Omega_0 + (\alpha - \alpha')Q^2 \\
\Omega_2(\mathbf{Q}) &= \Omega_0 + (\alpha - \alpha')Q^2 \\
\Omega_3(\mathbf{Q}) &= \Omega_0 + (\alpha + \alpha')Q^2 \\
\Omega_3(\mathbf{Q}) &= \Omega_0 + (\alpha + \alpha')Q^2 + 4A|Q|
\end{aligned} \tag{3}$$

Thus, we clearly see that there should be two degenerate parabolic bands (Ω_1 and Ω_2), another parabolic band with a smaller effective mass (Ω_3), and a linear v-shaped band (Ω_4) in excellent agreement with the *ab initio* GW-BSE results.

In fact, for any centro-symmetric system with N-fold degenerate excitons at $\mathbf{Q} = 0$, if we can assume that the exchange matrix elements between all the degenerate excitons are approximately equal, then there will always be $N - 1$ parabolic bands and 1 linearly dispersing band because the exchange matrix is proportional to a matrix-of-ones.

B. Few-layer Black Phosphorus

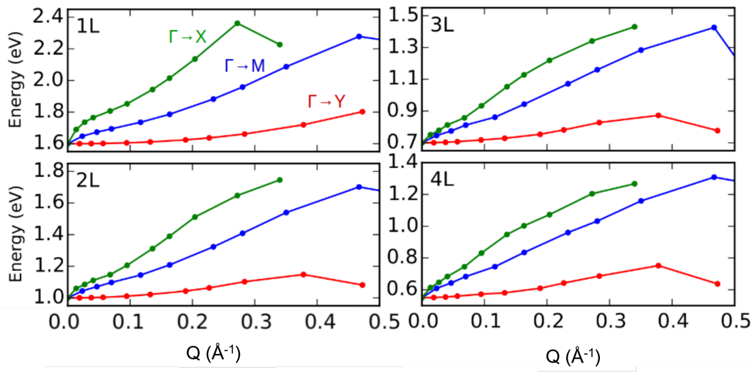

FIG. S3. Exciton bandstructure of 1 layer (1L) through 4 layer (4L) black phosphorus calculated within GW-BSE.

III. DERIVATION OF EFFECTIVE HAMILTONIAN FOR SMALL Q

Electron-hole interactions are built on top of the quasiparticle (QP) picture by solving the BSE in the electron-hole basis[5,21,28]:

$$(E_{c\mathbf{k}+\mathbf{Q}} - E_{v\mathbf{k}})\, A^S_{vc\mathbf{k}\mathbf{Q}} + \sum_{v'c'\mathbf{k}'} \langle v\mathbf{k}; c\mathbf{k}+\mathbf{Q}| K^{eh} \,|v'\mathbf{k}'; c'\mathbf{k}'+\mathbf{Q}\rangle\, A^S_{v'c'\mathbf{k}'\mathbf{Q}} = \Omega^S_{\mathbf{Q}} A^S_{vc\mathbf{k}\mathbf{Q}}. \quad (4)$$

Here, the index $(v\mathbf{k}; c\mathbf{k}+\mathbf{Q})$ indicates a hole state $|v\mathbf{k}\rangle$ and an electron state $|c\mathbf{k}+\mathbf{Q}\rangle$, where $\mathbf{k}$ is the crystal momentum and $\mathbf{Q}$ is the exciton center-of-mass momentum; $E_{c\mathbf{k}+\mathbf{Q}}$ and $E_{v\mathbf{k}}$ are the QP energies calculated within the GW approximation; S indexes the exciton state at momentum $\mathbf{Q}$; $A^S_{v\mathbf{k};c\mathbf{k}+\mathbf{Q}}$ is the amplitude of the free electron-hole pair; $\Omega^S_{\mathbf{Q}}$ is the exciton excitation energy; and K^{eh} is the electron-hole interaction kernel. To obtain the exciton bandstructure (or dispersion), we solve the BSE at different exciton momenta $\mathbf{Q}$ following the methodology developed in Ref.[6] and [17].

The first term in Eq. 4, $(E_{c\mathbf{k}+\mathbf{Q}} - E_{v\mathbf{k}})$, is the electron-hole transition energy, analogous to the kinetic energy. When the electron-hole interaction is small, this term dominates, and as long as the electron and hole each arise from a single parabolic band, the exciton dispersion is parabolic with the mass of the exciton equal to the sum of the electron and hole band masses. This is the limit where the commonly used effective mass approximation holds. The interaction kernel in Eq. 4 is

$K^{eh} = K^d + K^x$ (or $K^{eh} = K^d + 2K^x$, when the spin-orbit interaction is neglected); it consists of a direct term (K^d), where the attractive interaction between the electron and the hole is mediated by the screened Coulomb interaction,

$$\begin{aligned} &\langle v\mathbf{k}; c\mathbf{k}+\mathbf{Q}| K^d |v'\mathbf{k}'; c'\mathbf{k}'+\mathbf{Q}\rangle \\ &= -\sum_{\mathbf{GG}'} M^*_{cc'}(\mathbf{k}+\mathbf{Q},\mathbf{q},\mathbf{G}) W_{\mathbf{GG}'}(\mathbf{q}) M_{vv'}(\mathbf{k},\mathbf{q},\mathbf{G'}), \end{aligned} \tag{5}$$

and an exchange term (K^x), where the exchange scattering of an electron-hole pair gives rise to a repulsive term mediated by the bare Coulomb interaction,

$$\begin{aligned} &\langle v\mathbf{k}; c\mathbf{k}+\mathbf{Q}| K^x |v'\mathbf{k}'; c'\mathbf{k}'+\mathbf{Q}\rangle \\ &= \sum_{\mathbf{G}} M^*_{cv}(\mathbf{k},\mathbf{Q},\mathbf{G}) \mathrm{v}(\mathbf{Q}+\mathbf{G}) M_{c'v'}(\mathbf{k}',\mathbf{Q},\mathbf{G}). \end{aligned} \tag{6}$$

Here, W and v are the screened and the bare Coulomb interactions, respectively; $\mathbf{G}$ are the reciprocal lattice vectors; $\mathbf{q} = \mathbf{k} - \mathbf{k}'$; and M are the plane-wave matrix elements such that $M_{nn'}(\mathbf{k},\mathbf{q},\mathbf{G}) = \langle n\mathbf{k}|e^{i(\mathbf{q}+\mathbf{G})\cdot\mathbf{r}}|n'\mathbf{k}'\rangle$[5,20]. It is important to note that in typical calculations of optical spectra within the BSE, the long-range (i.e. $\mathbf{G} = 0$) term in Eq. **??** is neglected[5,6,14] in order to avoid the non-analytic behavior at $\mathbf{Q} = 0$. This is equivalent to solving for only transverse excitons at exactly $\mathbf{Q} = 0$[3] but leads to spurious results at finite momentum. Thus, we solve the BSE including both long and short-range exchange, and the resulting exciton bandstructure contains *both* longitudinal and transverse solutions.

We derive here the contribution of the long-range (**G**=0) exchange to the effective Hamiltonian in 1D, 2D, and 3D. To derive an effective Hamiltonian, we separate the BSE Hamiltonian into two contributions at $\mathbf{Q} = 0$ and finite $\mathbf{Q}$.

$$\begin{aligned} H^{BSE}(\mathbf{Q}) &= H^{BSE}(\mathbf{0}) + [H^{BSE}(\mathbf{Q}) - H^{BSE}(\mathbf{0})] \\ &= H^{BSE}(\mathbf{0}) + \frac{\hbar^2 Q^2}{2M} + [K^x(\mathbf{Q}) - K^x(\mathbf{0})] + [K^d(\mathbf{Q}) - K^d(\mathbf{0})], \end{aligned} \tag{7}$$

where K^x and K^d are the exchange and direct terms and $M = m_e + m_h$ is the band mass of the free electron-hole pair at the band extrema in each material system. We will write the Hamiltonian in the basis of finite momentum exciton states $|S(\mathbf{Q})\rangle$. $H^{BSE}(\mathbf{0})$ is a diagonal matrix in the basis of exciton states with the eigenvalues of the exciton states at $\mathbf{Q} = 0$ ($\Omega_{\mathbf{0}}$) along the diagonal.

Next, we determine the **Q**-dependent terms of the exchange interaction, $[K^x(\mathbf{Q}) - K^x(\mathbf{0})]$. Here, the spin-index is included implicitly in the valence and conduction band indices (v and c), and the exchange term is only non-zero for S=0 excitons.

We perform a $\mathbf{Q} \cdot \mathbf{p}$ expansion of the quasiparticle (QP) states up to second order. M becomes

$$\begin{aligned} M_{cv}(\mathbf{k},\mathbf{Q},\mathbf{G}) =& \langle u_{c\mathbf{k}}|e^{i\mathbf{G}\cdot\mathbf{r}}|u_{v\mathbf{k}}\rangle + \mathbf{Q}\cdot\frac{\hbar}{m}\sum_{n\neq c}\frac{(\langle u_{n\mathbf{k}}|\mathbf{p}|u_{c\mathbf{k}}\rangle)^*}{E_{c\mathbf{k}}-E_{n\mathbf{k}}}\langle u_{n\mathbf{k}}|e^{i\mathbf{Q}+\mathbf{G}\cdot\mathbf{r}}|u_{v\mathbf{k}}\rangle \\ &+ \left(\frac{\hbar}{m}\right)^2\sum_{n\neq c}\sum_{m\neq c}\frac{[(\mathbf{Q}\cdot\langle u_{n\mathbf{k}}|\mathbf{p}|u_{m\mathbf{k}}\rangle)(\mathbf{Q}\cdot\langle u_{m\mathbf{k}}|\mathbf{p}|u_{c\mathbf{k}}\rangle)]^*}{(E_{c\mathbf{k}}-E_{n\mathbf{k}})(E_{c\mathbf{k}}-E_{m\mathbf{k}})}\langle u_{n\mathbf{k}}|e^{i\mathbf{G}\cdot\mathbf{r}}|u_{v\mathbf{k}}\rangle \end{aligned} \tag{8}$$

where $|u_{c\mathbf{k}}\rangle$ and $|u_{n\mathbf{k}}\rangle$ are the periodic parts of the Bloch wavefunctions.

Keeping terms up to lowest order in $\mathbf{Q}$, the long-range ($\mathbf{G}=0$) term becomes

$$M_{cv}(\mathbf{k},\mathbf{Q},\mathbf{G}=0) = \mathbf{Q}\cdot\frac{\hbar}{m}\sum_{n\neq c}\frac{(\langle u_{n\mathbf{k}}|\mathbf{p}|u_{c\mathbf{k}}\rangle)^*}{E_{c\mathbf{k}}-E_{n\mathbf{k}}}\langle u_{n\mathbf{k}}|u_{v\mathbf{k}}\rangle + \mathcal{O}(\mathbf{Q}^2) \tag{9}$$

Eq. 9 simplifies to

$$M_{cv}(\mathbf{k},\mathbf{Q},\mathbf{G}=0) = \mathbf{Q}\cdot\frac{\hbar}{m(E_{c\mathbf{k}}-E_{v\mathbf{k}})}\langle u_{c\mathbf{k}}|\mathbf{p}|u_{v\mathbf{k}}\rangle + \mathcal{O}(\mathbf{Q}^2) \tag{10}$$

Substituting Eq. 10 into Eq. 6, the long-range ($\mathbf{G}=0$) exchange becomes

$$\begin{aligned} \langle vc\mathbf{kQ}|K^x_{LR}|v'c'\mathbf{k'Q}\rangle &= \frac{\hbar^2}{m^2(E_{c\mathbf{k}}-E_{v\mathbf{k}})(E_{c'\mathbf{k}}-E_{v'\mathbf{k}})}(\mathbf{Q}\cdot\langle u_{c\mathbf{k}}|\mathbf{p}|u_{v\mathbf{k}}\rangle)(\mathbf{Q}\cdot\langle u'_{v'\mathbf{k}}|\mathbf{p}|u_{c'\mathbf{k'}}\rangle v(\mathbf{Q})) \\ &= a|\mathbf{Q}|^2\cos(\phi_\mathbf{Q})\cos(\phi'_\mathbf{Q})v(\mathbf{Q}), \end{aligned} \tag{11}$$

where $a = \frac{\hbar^2}{m^2(E_{c\mathbf{k}}-E_{v\mathbf{k}})(E_{c'\mathbf{k}}-E_{v'\mathbf{k}})}|\langle u_{c\mathbf{k}}|\mathbf{p}|u_{v\mathbf{k}}\rangle)|\times|\langle u'_{v'\mathbf{k}}|\mathbf{p}|u_{c'\mathbf{k'}}\rangle|$ and $\phi_\mathbf{Q}$ is the angle between $\mathbf{Q}$ and $\langle u_{c\mathbf{k}}|\mathbf{p}|u_{v\mathbf{k}}\rangle$.

In the exciton basis, the long-range exchange is

$$\begin{aligned} \langle S(\mathbf{Q})|K^x_{LR}|S'(\mathbf{Q})\rangle &= \sum_{vc\mathbf{k},v'c'\mathbf{k'}} A^{S*}_{vc\mathbf{k}}A^{S'}_{v'c'\mathbf{k'}}\langle vc\mathbf{kQ}|K^x_{LR}|v'c'\mathbf{k'Q}\rangle \\ &= \frac{\hbar^2}{m^2}(\mathbf{Q}\cdot\sum_{vc\mathbf{k}}A^{S*}_{vc\mathbf{k}}(E_{c\mathbf{k}}-E_{v\mathbf{k}})^{-1}\langle u_{c\mathbf{k}}|\mathbf{p}|u_{v\mathbf{k}}\rangle)(\mathbf{Q}\cdot\sum_{v'c'\mathbf{k'}}A^{S'*}_{v'c'\mathbf{k'}}(E_{c'\mathbf{k'}}-E_{v'\mathbf{k'}})^{-1}\langle u'_{v'\mathbf{k}}|\mathbf{p}|u_{c'\mathbf{k'}}\rangle v(\mathbf{Q}) \\ &\propto (\mathbf{Q}\cdot(\langle 0|\mathbf{r}|S\rangle)^*)(\mathbf{Q}\cdot\langle 0|\mathbf{r}|S'\rangle)v(\mathbf{Q}) \\ &= \tilde{a}|\mathbf{Q}|^2\cos(\theta_\mathbf{Q})\cos(\theta'_\mathbf{Q})v(\mathbf{Q}), \end{aligned} \tag{12}$$

where $\tilde{a} = |\langle 0|\mathbf{r}|S\rangle| \times |\langle 0|\mathbf{r}|S'\rangle|$ and $\theta_\mathbf{Q}$ is the angle between $\mathbf{Q}$ and $\langle 0|\mathbf{r}|S\rangle$. Note, to evaluate $\langle 0|\mathbf{r}|S\rangle$, we use the dipole approximation $i\mathbf{Q}\cdot\mathbf{r} \approx e^{i\mathbf{Q}\cdot\mathbf{r}} - 1$, following the evaluation of optical matrix elements in BerkeleyGW[5].

The Coulomb interaction ($v(\mathbf{Q})$) in reciprocal space approaches different limits depending on the dimensionality[13,17]:

$$\mathrm{v}(\mathbf{Q}\rightarrow 0)=\begin{array}{cc} \frac{1}{Q^2} & \text{in 3D} \\ \frac{1}{|Q|} & \text{in 2D} \\ -2(\gamma_E+\ln|Q|) & \text{in 1D} \end{array}, \tag{13}$$

where $\gamma_E = 0.577$ is the Euler constant. Applying the limit of $v(\mathbf{Q})$ and switching to the exciton basis, for which K^x is diagonal for non-degenerate excitons, the small $\mathbf{Q}$ (or long wavelength) limit of the exchange term is

$$\langle S(\mathbf{Q})|K^x_{LR}|S(\mathbf{Q})\rangle=\begin{array}{cc} \alpha\cos^2(\theta_{\mathbf{Q}}) & \text{in 3D} \\ \alpha|\mathbf{Q}|\cos^2(\theta_{\mathbf{Q}}) & \text{in 2D} \\ -2\alpha|\mathbf{Q}|^2\cos^2(\theta_{\mathbf{Q}})(\gamma_E+\ln|Q|) & \text{in 1D} \end{array}, \tag{14}$$

were $\alpha=\frac{\hbar^2}{m^2}|\sum_{v c\mathbf{k}}(E_{c\mathbf{k}}-E_{v\mathbf{k}})^{-1}A^S_{vc\mathbf{k}}\langle c\mathbf{k}|\mathbf{p}|v\mathbf{k}\rangle|^2$ and $\theta_{\mathbf{Q}}$ is the angle between $\mathbf{Q}$ and $\langle 0|\mathbf{p}|S(\mathbf{Q})\rangle$.

For the other terms in Eq. 7, the derivation of the small-$\mathbf{Q}$ behavior for both K^d in the supplemental information of Ref.[17], where it shown that $K^d(\mathbf{Q})-K^d(0)\propto Q^2$. Likewise, the derivation of both long-range and short-range terms for $K^x(\mathbf{Q})-K^x(0)$ can be found in the supplemental information of Ref.[17], where it is shown that the short-range exchange also has a Q^2 dependence. Thus, in constructing the model Hamiltonian, we combine a constant term representing the excitation energy of the transverse exciton at $\mathbf{Q}$=0 (which we will call Ω_0) with the dimensional-dependent long-range exchange term (Eq. 10), and an $O(\mathbf{Q}^2)$ term that captures the higher-order $\mathbf{Q}$-dependent contributions of the direct and exchange interaction. The $O(\mathbf{Q}^2)$ term can be fit to obtain an effective mass, so in all cases we write it as $\frac{\hbar^2 Q^2}{2M^*}$, where M^* is the exciton effective mass in the limit of zero long-range exchange. We note that an exciton effective mass cannot be defined when the long-range exchange is non-zero, since the long-range exchange always introduces a term that is non-analytic at $\mathbf{Q}$=0.

Combining all terms, in the exciton basis, the model Hamiltonian for 3D materials has the general form

$$H^{\mathrm{BSE}}(\mathbf{Q})=\Omega_0+C\cos^2(\theta_{\mathbf{Q}})+\frac{\hbar^2}{2}(\frac{Q_x^2}{M_x^*}+\frac{Q_y^2}{M_y^*}+\frac{Q_z^2}{M_z^*}), \tag{15}$$

where C is a constant that we fit to the computed *ab initio* exciton bandstructure. We note that M^* is not equivalent to the sum of the electron and hole quasiparticle band masses because the

electron-hole interaction introduces a mass enhancement. We obtain M^* by fitting to the computed *ab initio* exciton bandstructure (see Table 1 below).

As an example 3D system, we study crystal pentacene. For the bright excitons, the long-range exchange is

$$\langle S_B(\mathbf{Q})|K^x_{LR}|S_B(\mathbf{Q})\rangle = C\cos^2(\theta_{\mathbf{Q}}), \tag{16}$$

where the angle $\theta_{\mathbf{Q}}$ is measured with respect to Γ to X axis. For the dark (dipole-forbidden) excitons, the long-range exchange is

$$\langle S_B(\mathbf{Q})|K^x_{LR}|S_B(\mathbf{Q})\rangle = 0. \tag{17}$$

The model Hamiltonian for 2D materials has the general form

$$H^{\mathrm{BSE}}(\mathbf{Q}) = \Omega_0 + A|Q|\cos^2(\theta_{\mathbf{Q}}) + \frac{\hbar^2}{2}(\frac{Q_x^2}{M_x^*} + \frac{Q_y^2}{M_y^*}), \tag{18}$$

where A is a constant that we fit to the *ab initio* exciton bandstructure and M^* is the exciton effective mass. We note that M^* is not equivalent to the sum of the electron and hole quasiparticle band masses because the electron-hole interaction introduces a mass enhancement. We obtain M^* by fitting to the *ab initio* exciton bandstructure (see Table 1 below). For excitons that are degenerate at $\mathbf{Q} = 0$, $H^{\mathrm{BSE}}(\mathbf{Q})$ can be written as a matrix in the basis of degenerate exciton states at $\mathbf{Q} = 0$. The matrix is diagonalized to obtain finite-momentum solutions. This is demonstrated for the case of MoS_2 in Ref. [17].

The model Hamiltonian for 1D materials has the general form

$$H^{\mathrm{BSE}}(\mathbf{Q}) = \Omega_0 + B|\mathbf{Q}|^2\cos^2(\theta_{\mathbf{Q}})(\gamma_E + \ln|Q|)) + \frac{\hbar^2 Q^2}{2M^*}, \tag{19}$$

where B is a constant that we fit to the *ab initio* exciton bandstructure. We obtain M^* by fitting to the computed *ab initio* exciton bandstructure (see Table 1 below). We note that it is not equivalent to the sum of the electron and hole quasiparticle band masses due to a mass enhancement introduced by the electron-hole interaction.

IV. LONGITUDINAL AND TRANSVERSE EXCITONS IN THE BSE KERNEL

We note that in BSE calculations at zero momentum it is common to neglect the long-range exchange term (Eq. 10) to avoid the non-analytic behavior of $\mathrm{v}(\mathbf{Q})$ at $\mathbf{Q} = 0$. This is equivalent to calculating *only* transverse exciton states at $\mathbf{Q} = 0$. However, if this term is neglected for

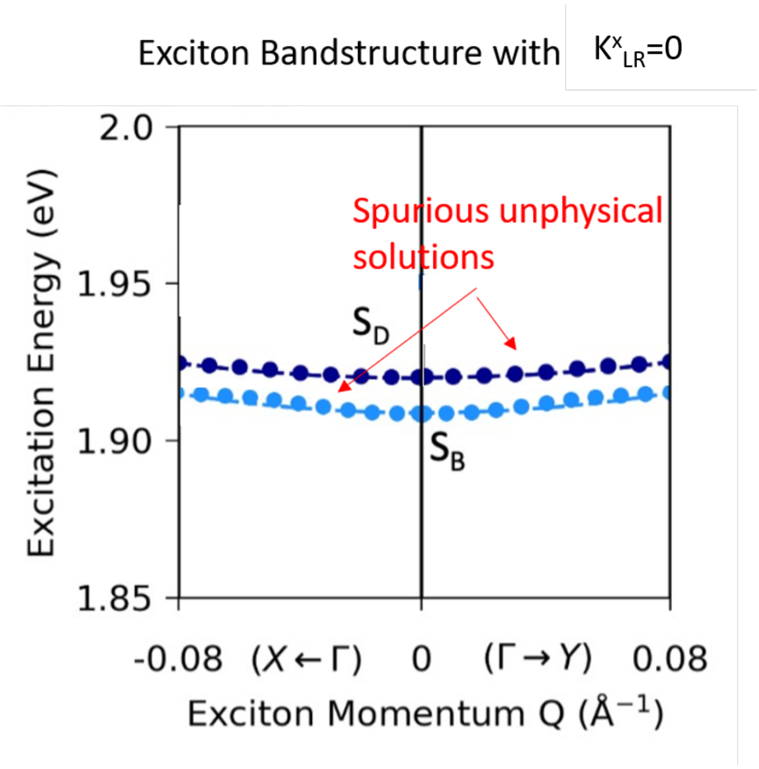

FIG. S4. Exciton band structure of crystal pentacene calculated when the long-range exchange is neglected in the BSE Hamiltonian. The bright exciton branch along $\Gamma \rightarrow X$, and the dark exciton branch along $\Gamma \rightarrow Y$ are unphysical solutions.

finite-momentum excitons, the resulting exciton bandstructure is *not* the bandstructure of transverse excitons. Instead, the bandstructure will contain the dispersion of transverse exciton states combined with spurious unphysical solutions. These unphysical solutions appear because the solution of the BSE does not explicitly contain the polairzation of the external field, so there is no way to restrict the BSE to solve for only transverse excitons. The unphysical solutions may be interpreted as longitudinal excitons, where the long-range exchange is neglected. For demonstration, Fig. S4 shows the exciton band structure of crystal pentacene when the long-range exchange is neglected.

TABLE I. GW-BSE exciton properties, extracted from the exciton bandstructures and used for the computed exciton propagation, and fit to the extended model Hamiltonians derived in this work for different dimensions.

System	State	$\Omega(0)$[eV]	m_h	m_e	$m_h + m_e$	M^*	Fit of $\Omega(\mathbf{Q})$
Bulk Pentacene	S_B(X)	1.95	4.6	7.6	12.2	15.4	$\Omega(Q)_X = \Omega(0)_Y + 0.04 + \frac{\hbar^2}{2M_x^*}Q^2$
	S_B(Y)	1.91	2.2	1.6	3.8	4.1	$\Omega(Q)_Y = \Omega(0)_Y + \frac{\hbar^2}{2M_y^*}Q^2$
	S_D(X)	1.92	4.6	7.6	12.2	5.2	$\Omega(Q)_X = \Omega(0)_Y + \frac{\hbar^2}{2M_x^*}Q^2$
	S_D(Y)	1.92	2.2	1.6	3.8	4.6	$\Omega(Q)_Y = \Omega(0)_Y + \frac{\hbar^2}{2M_y^*}Q^2$
ML MoS_2	S_P	2.05	0.6	0.5	1.1	1.4	$\Omega(Q) = \Omega(0) + \frac{\hbar^2}{2M^*}Q^2$
	S_L	2.05	0.6	0.5	1.1	–	$\Omega(Q) = \Omega(0) + 1.418\|Q\|$
Black Phosphorus	S_B(X)	1.59	0.3	0.3	0.60	–	$\Omega(Q)_X = \Omega(0)_X + 8.00\|Q\|$
	S_B(Y)	1.59	3.9	0.7	4.6	20	$\Omega(Q)_Y = \Omega(0)_Y + \frac{\hbar^2}{2M_y^*}Q^2$
SWCNT (0,8)	S_B	1.48	0.3	0.2	0.5	–	$\Omega(Q) = \Omega(0) - 52Q^2(\gamma_E^* + ln\|Q\|)$
	S_{D1}	1.44	0.3	0.2	0.5	0.4	$\Omega(Q) = \Omega(0) + \frac{\hbar^2}{2M^*}Q^2$
	S_{D2}	1.52	0.3	0.2	0.5	0.6	$\Omega(Q) = \Omega(0) + \frac{\hbar^2}{2M^*}Q^2$

V. EXCITON DISPERSION FUNCTIONS AND EFFECTIVE MASSES

A. GW-BSE exciton dispersion and effective masses

Table I shows the computed GW-BSE exciton properties: the $\mathbf{Q} = 0$ excitation energy $\Omega(0)$; the electron (m_e) and hole (m_h) effective masses, and the corresponding exciton effective mass within the effective mass model, $M^{\text{eff}} = m_h + m_e$; the exciton effective mass M^*, taken explicitly from the exciton dispersion around $\mathbf{Q} = 0$ for the analytic dipole forbidden exciton bands (an effective mass cannot be defined for the non-analytic bands); and the fit of the exciton dispersion to the solution of the model Hamiltonian for each material along the examined dispersion directions.

B. Solid pentacene anisotropy in exciton dispersion

In the main text, we present the exciton dispersion of solid pentacene at the $\mathbf{a}, \mathbf{b}$-directions around $\mathbf{Q} = 0$. Fig. S5 shows the exciton dispersion at larger values of $\mathbf{Q}$ and at all main crystal

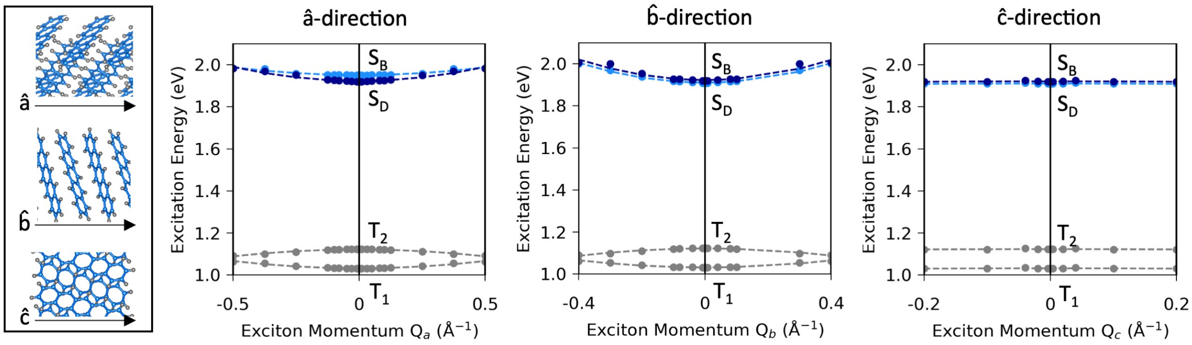

FIG. S5. Exciton bandstructure of solid pentacene, at the $\hat{a}$, $\hat{b}$, and $\hat{c}$ unit cell directions.

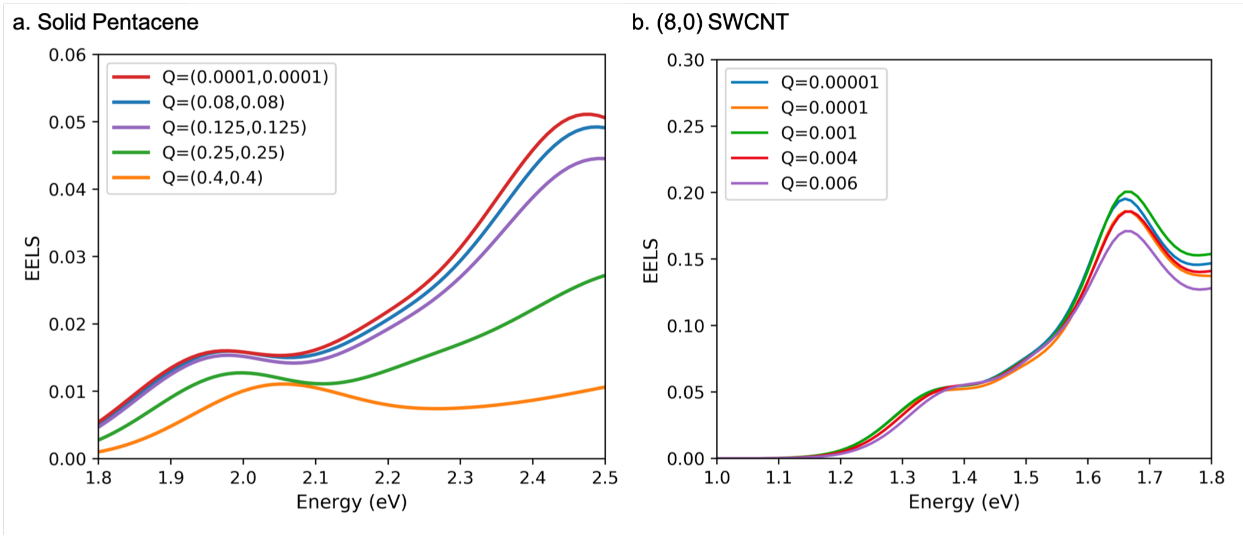

FIG. S6. EELS spectra of (a) pentacene molecular crystal; (b) (8,0) single-walled carbon nanotube. The finite momentum $\mathbf{Q}$ is directed along the $\hat{a}+\hat{b}$ direction in the pentacene crystal and the $\hat{c}$ direction in the SWCNT.

directions, to emphasize that the dispersion is 2D-like due to the crystal packing and that the excitations are anisotropic in this quasi-plane.

VI. ELECTRON ENERGY-LOSS SPECTRA

The computed band dispersion can be directly related to measured EELS spectra. In fig.S6 we show the calculated energy-loss spectra of pentacene crystal and of (8,0) SWCNT, via $\epsilon_2/[\epsilon_1^2+\epsilon_2^2]$.

ϵ_2 is directly computed from BSE; for pentacene ϵ_1 is shifted so that at $E = 0$ it corresponds to the computed GW dielectric constant. In both cases, the spectra compares well with related experiment[11,22–24]. For example, the pentacene crystal spectra for the range of finite exciton momentum follows the trend observed in experiment, with the increase in momentum the first peak is blue-shifted and the intensity decreases[11,22,24]. Notably, for SWCNT the available experimental electron energy-loss spectroscopy is for different structures than the one computed here [23].

VII. EXCITON WAVEPACKET TIME EVOLUTION

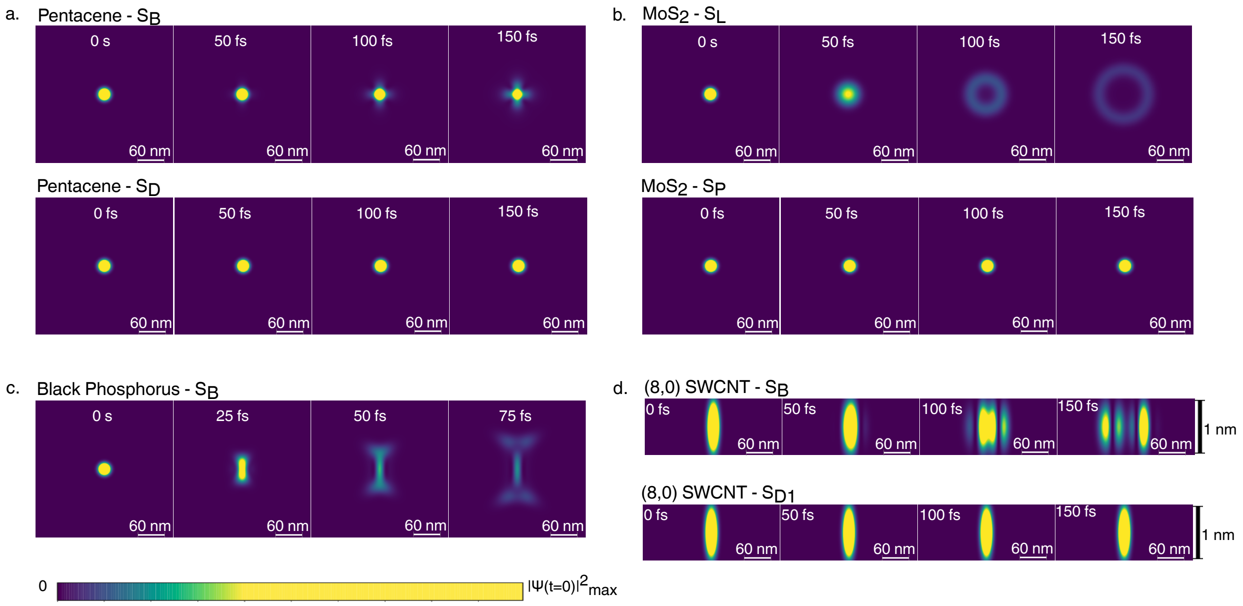

FIG. S7. Time evolution of the amplitude squared ($|\Psi(\mathbf{R}, t)|^2$) of an initial Gaussian exciton wavepacket as a function of the bandstructure of a single exciton band, as labeled in Fig.1 in the main text, for a) pentacene, b) monolayer MoS_2, c) monolayer black phosphorus, and d) the (8,0) SWCNT. The initial wavepacket at $t = 0$ has the same spatial distribution in all cases, but the different band dispersion leads to distinct features in the wavepacket evolution with time. In all cases, the distribution is normalized with respect to the maximum value at time $t = 0$.

.

[*] diana.qiu@yale.edu

[†] sivan.refaely-abramson@weizmann.ac.il

[1] https://summary.ccdc.cam.ac.uk.

[2] Felipe H. da Jornada, Diana Y. Qiu, and Steven G. Louie. Nonuniform sampling schemes of the brillouin zone for many-electron perturbation-theory calculations in reduced dimensionality. *Phys. Rev. B*, 95:035109, 2017.

[3] M. M. Denisov and V. P. Makarov. Longitudinal and transverse excitons in semiconductors. *Phys. Status Solidi B*, 56(1):9–59, 1973.

[4] Jack Deslippe, Georgy Samsonidze, Manish Jain, Marvin L. Cohen, and Steven G. Louie. Coulomb-hole summations and energies for gw calculations with limited number of empty orbitals: a modified static remainder approach. *Phys. Rev. B.*, 87:165124, 2013.

[5] Jack Deslippe, Georgy Samsonidze, David A. Strubbe, Manish Jain, Marvin L. Cohen, and Steven G. Louie. Berkeleygw: A massively parallel computer package for the calculation of the quasiparticle and optical properties of materials and nanostructures. *Comput. Phys. Commun.*, 183(6):1269 – 1289, 2012.

[6] Matteo Gatti and Francesco Sottile. Exciton dispersion from first principles. *Phys. Rev. B*, 88:155113, Oct 2013.

[7] P. Giannozzi et al. Quantum espresso: a modular and open-source software project for quantum simulations of materials. *J. Phys.: Condens. Matter*, 21:395502, 2009.

[8] Stefan Grimme. Semiempirical gga-type density functional constructed with a long-range dispersion correction. *J. Comput. Chem.*, 27(15):1787–1799, 2006.

[9] M. S. Hybertsen and S. G. Louie. Electron correlation in semiconductors and insulators: Band gaps and quasiparticle energies. *Phys. Rev. B*, 34:5390, 1986.

[10] Sohrab Ismail-Beigi. Truncation of periodic image interactions for confined systems. *Phys. Rev. B*, 73:233103, 2006.

[11] M Knupfer and H Berger. Dispersion of electron–hole excitations in pentacene along (1 0 0). *Chemical physics*, 325(1):92–98, 2006.

[12] W. Kohn and L. J. Sham. Self-consistent equations including exchange and correlation effects. *Phys. Rev.*, 140:A1133–A1138, 1965.

[13] Bogdan Mihaila. Lindhard function of a d-dimensional fermi gas. *arXiv preprint arXiv:1111.5337*, 2011. https://arxiv.org/abs/1111.5337.

[14] Giovanni Onida, Lucia Reining, and Angel Rubio. Electronic excitations: density-functional versus many-body green's-function approaches. *Rev. Mod. Phys.*, 74:601–659, Jun 2002.

[15] John P. Perdew, Kieron Burke, and Matthias Ernzerhof. Generalized gradient approximation made simple. *Phys. Rev. Lett.*, 77:3865, 1996.

[16] John P. Perdew, Kieron Burke, and Matthias Ernzerhof. Generalized gradient approximation made simple. *Phys. Rev. Lett.*, 77:3865, 1996.

[17] D. Y. Qiu, T. Cao, and S. G. Louie. Nonanalyticity, valley quantum phases, and lightlike exciton dispersion in monolayer transition metal dichalcogenides: Theory and first-principles calculations. *Phys. Rev. Lett.*, 115:176801, 2015.

[18] Diana Y. Qiu, Felipe H. da Jornada, and Steven G. Louie. Environmental screening effects in 2d materials: Renormalization of the bandgap, electronic structure, and optical spectra of few-layer black phosphorus. *Nano Lett.*, 17:4706–4712, 2017.

[19] Tonatiuh Rangel, Kristian Berland, Sahar Sharifzadeh, Florian Brown-Altvater, Kyuho Lee, Per Hyldgaard, Leeor Kronik, and Jeffrey B. Neaton. Structural and excited-state properties of oligoacene crystals from first principles. *Phys. Rev. B*, 93:115206, 2016.

[20] Michael Rohlfing and S. G. Louie. Electron-hole excitations in semiconductors and insulators. *Phys. Rev. Lett.*, 81:2312, 1998.

[21] Michael Rohlfing and S. G. Louie. Electron-hole excitations and optical spectra from first principles. *Phys. Rev. B*, 62:4927, 2000.

[22] Friedrich Roth, Roman Schuster, Andreas König, Martin Knupfer, and Helmuth Berger. Momentum dependence of the excitons in pentacene. *J. Chem. Phys.*, 136(20):204708, 2012.

[23] Yohei Sato and Masami Terauchi. High-energy resolution electron energy-loss spectroscopy study of interband transitions characteristic to single-walled carbon nanotubes. *Microscopy and Microanalysis*, 20(3):807–814, 2014.

[24] R. Schuster, M. Knupfer, and H. Berger. Exciton band structure of pentacene molecular solids: Breakdown of the frenkel exciton model. *Phys. Rev. Lett.*, 98:037402, Jan 2007.

[25] Sahar Sharifzadeh, Pierre Darancet, Leeor Kronik, and Jeffrey B. Neaton. Low-energy charge-transfer excitons in organic solids from first-principles: The case of pentacene. *J. Phys. Chem. Lett.*, 4:2197–2201, 2013.

[26] T. Siegrist, C. Besnard, S. Haas, M. Schiltz, P. Pattison, D. Chernyshov, B. Batlogg, and C. Kloc. A polymorph lost and found: The high-temperature crystal structure of pentacene. *Adv. Mater.*, 19:2079, 2007.

[27] Catalin D Spataru, Sohrab Ismail-Beigi, Lorin X Benedict, and Steven G Louie. Quasiparticle energies, excitonic effects and optical absorption spectra of small-diameter single-walled carbon nanotubes. *Appl. Phys. A*, 78(8):1129–1136, 2004.

[28] G. Strinati. Application of the Green's functions method to the study of the optical properties of semiconductors. *Riv. Nuovo Cimento*, 11:1, 1988.

[29] P A Young. Lattice parameter measurements on molybdenum disulphide. *J Phys. D Appl. Phys.*, 1(7):936–938, jul 1968.
